# Case for diagnosis. Diffuse ulcerated nodular lesions^☆^^☆☆^

**DOI:** 10.1016/j.abd.2019.09.021

**Published:** 2019-09-30

**Authors:** Paulo Henrique Teixeira Martins, Gabriela Dallagnese, Laura Luzzatto, Manuela Lima Dantas

**Affiliations:** a Department of Dermatology, Santa Casa de Misericórdia de Porto Alegre, Porto Alegre, RS, Brazil; b Department of Dermatology, Universidade Federal de Ciências da Saúde de Porto Alegre, Porto Alegre, RS, Brazil

**Keywords:** Histiocytosis, Inflammation, Neoplasms

## Abstract

Langerhans cell histiocytosis is a rare clonal proliferative disease, characterized by the infiltration of one or multiple organs by histiocytes. Due to the diversity of signs and symptoms, the diagnosis of this disease is often late. The estimated incidence in adults is one to two cases per million, but the disease is probably underdiagnosed in this population. This report presents a case of disseminated Langerhans cell histiocytosis. The authors highlight the most characteristic aspects of this rare and heterogeneous disease, which usually presents as a challenging clinical diagnosis.

## Case report

**Figure 1:**
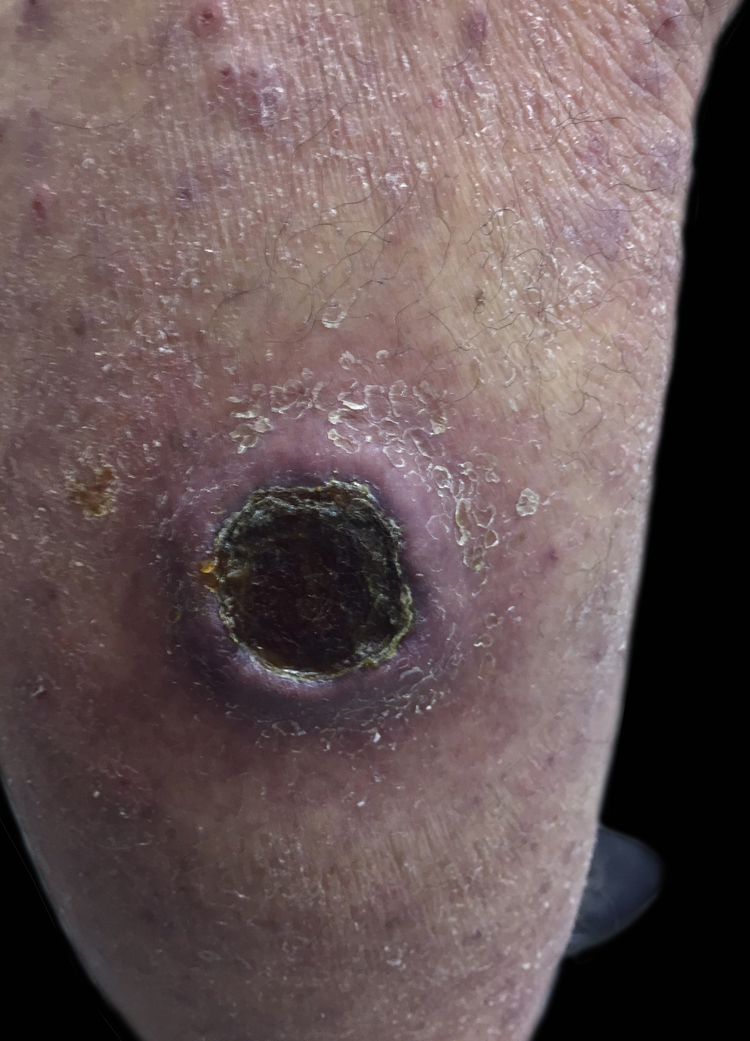
Ulcer with crust centers on the left lower limb.

**Figure 2:**
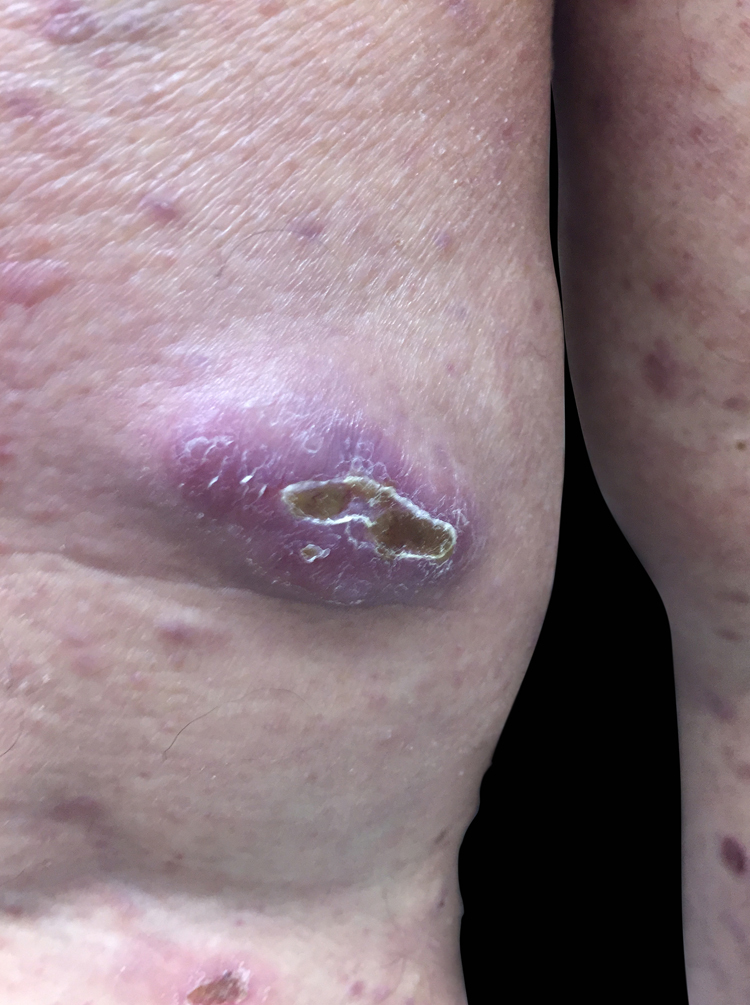
Nodular lesion on the right lower limb.

**Figure 3:**
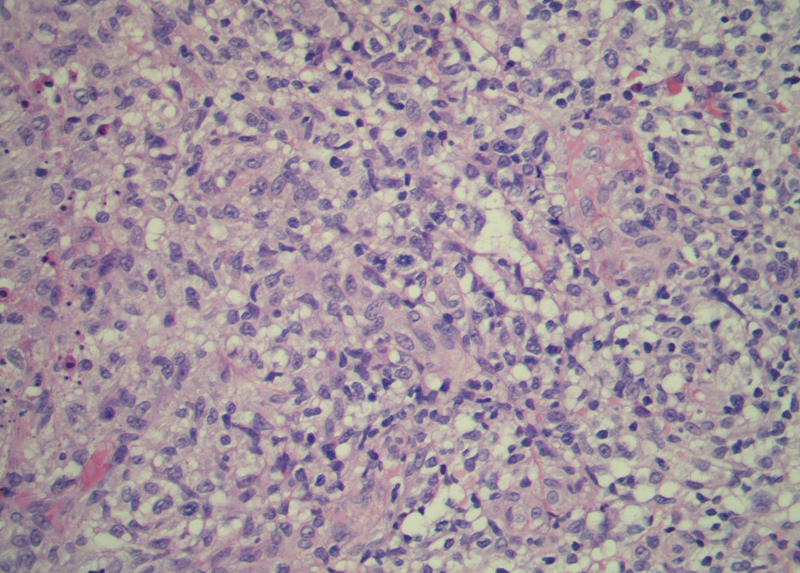
Histopathology with hematoxylin & eosin staining, ×40.

**Figure 4:**
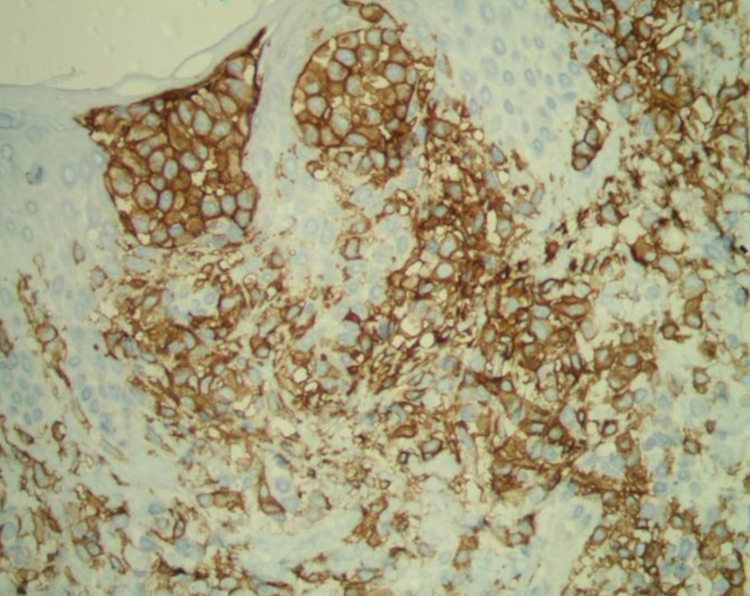
Immunohistochemistry – CD1a.

A female patient, 63 years old, had pruritic and diffused reddish spots on her body with about six months of evolution. Her external laboratory tests showed thrombocytopenia and anemia, and the anatomopathological exam suggested pharmacodermy. The physical examination showed multiple violaceous papules and nodules, sometimes with ulceration and crusting at both ends and lace-like erythematous spots in the abdomen (Figure 1, Figure 2). Skin biopsy was performed. The anatomopathological exam showed histiocytic infiltrate in the papillary and reticular dermis, forming cell aggregates of intermediate size, with clear and abundant cytoplasm, nuclei sometimes cleaved, and with pseudoclefts (Fig. 3). Immunohistochemistry showed immunoreactivity for S100, CD1a (Fig. 4), and langerin, suggesting, along with the anatomopathological exam and clinical history, Langerhans cell histiocytosis (LCH). The patient initiated systemic chemotherapy with vinblastine associated with prednisone. Due to little response after three cycles, the treatment was replaced with cytarabine. The patient died due to acute respiratory failure, likely due to pulmonary sepsis.

## Discussion

LCH is a rare and heterogeneous disease. With the recent discovery of the BRAF-V600E mutation in a high prevalence of LCH cases (50%–60%), the disease was recognized as cancer with marked inflammation.^1^, ^2^ Recent studies suggest a clinical correlation between the presence of the mutation and the recurrence and severity of the disease.^3^ There is a current division between local and disseminated LCH. The clinical manifestations vary widely due to differences between the age of onset, the proliferation rate of Langerhans cells, and the tissues and organs involved. Bone involvement is the most common form of presentation, both in adults and children. Skin rashes of this disease in adults can simulate other common dermatoses, such as seborrheic dermatitis and atopic eczema.^4^, ^5^ In this case, diffused erythematous lesions generated a clinical diagnosis of pharmacodermy. Cutaneous lesions are present in 40% of cases associated with the multisystem disease, thus their presence must motivate the investigation of other organs involved.^6^ The diagnosis requires a high index of suspicion and depends on clinical and radiology findings associated with histopathology and immunohistochemistry.^4^ The gold standard test verifies the presence of Birbeck bodies, granules in the cytoplasm of Langerhans cells, in the electron microscopy. The main immunohistochemical manifestation is the presence of the proteins S100 and CD1a(+).^6^, ^7^ The treatment must be individualized, considering the organs infected, the disease extent, and the age group affected.^7^ Surgery, intralesional corticotherapy, and local radiotherapy are some of the therapeutic options for the local disease. In case of multisystem disease or involvement of risk organs (spleen, liver, bone marrow, and lung), chemotherapy is indicated (vinblastine and prednisolone, cytarabine, among others).^2^, ^6^ BRAF inhibitors such as vemurafenib are new therapeutic options.^2^ Although the therapy improves the survival rate, morbidity remains high for patients with Langerhans cells histiocytosis, and permanent sequelae are observed in 20%–30% of patients.^8^ The treatment of this condition needs to be provided in specialized centers in order to provide multidisciplinary care.^3^, ^7^

## Financial Support

None declared.

## Author's contribution

*Paulo Henrique Teixeira Martins*: Statistical analysis; approval of the final version of the manuscript; conception and planning of the study; elaboration and writing of the manuscript; obtaining, analyzing and interpreting the data; intellectual participation in propaedeutic and/or therapeutic conduct of the cases studied; critical review of the literature; critical review of the manuscript.

*Gabriela Dallagnese*: Elaboration and writing of the manuscript; critical review of the literature; critical review of the manuscript.

*Laura Luzzatto*: Effective participation in research orientation.

*Manuela Lima Dantas*: Elaboration and writing of the manuscript; critical review of the literature; critical review of the manuscript.

## Conflicts of interest

None declared.
